# Feasibility of wearable monitors to detect heart rate variability in children with hand, foot and mouth disease

**DOI:** 10.1186/s12879-024-08994-x

**Published:** 2024-02-15

**Authors:** Le Nguyen Thanh Nhan, Nguyen Thanh Hung, Truong Huu Khanh, Nguyen Thi Thu Hong, Nguyen Thi Han Ny, Le Nguyen Truc Nhu, Do Duong Kim Han, Tingting Zhu, Tran Tan Thanh, Girmaw Abebe Tadesse, David Clifton, H. Rogier Van Doorn, Le Van Tan, C. Louise Thwaites

**Affiliations:** 1 Children’s Hospital Number 1, Ho Chi Minh City, Vietnam; 2 Oxford University Clinical Research Unit, Ho Chi Minh City, Vietnam; 3 Department of Engineering Science, University of Oxford, Oxford, UK; 4 Centre for Tropical Medicine and Global Health, University of Oxford, Oxford, UK

**Keywords:** Hand foot and mouth disease, Vietnam, Wearable devices, Heart rate variability

## Abstract

Hand foot and mouth disease (HFMD) is caused by a variety of enteroviruses, and occurs in large outbreaks in which a small proportion of children deteriorate rapidly with cardiopulmonary failure. Determining which children are likely to deteriorate is difficult and health systems may become overloaded during outbreaks as many children require hospitalization for monitoring. Heart rate variability (HRV) may help distinguish those with more severe diseases but requires simple scalable methods to collect ECG data.

We carried out a prospective observational study to examine the feasibility of using wearable devices to measure HRV in 142 children admitted with HFMD at a children’s hospital in Vietnam. ECG data were collected in all children. HRV indices calculated were lower in those with enterovirus A71 associated HFMD compared to those with other viral pathogens.

HRV analysis collected from wearable devices is feasible in a low and middle income country (LMIC) and may help classify disease severity in HFMD.

**Supplementary Information:**

The online version contains supplementary material available at 10.1186/s12879-024-08994-x.

## Introduction

Hand foot and mouth disease (HFMD) is caused by enteroviruses and mainly affects children under five years of age. Over the last two decades, HFMD has become a major public health concern in the Asia–Pacific region where huge outbreaks have occurred. To date, these outbreaks total more than 16,000,000 cases and 4000 deaths [1, 2]. Over recent years, major outbreaks of HFMD have occurred in Vietnam: over 200,000 children were hospitalized in 2011–12 and over 130,000 in 2018 [3]. Whilst enterovirus A71 (EV-A71) remains the dominant enterovirus associated with severe HFMD in Vietnam,, cocksackievirus A10 (CVA10), cocksackievirus A6 (CVA6), and cocksackievirus A8 (CVA8) have also been detected [3].

Whilst infections are generally asymptomatic or uncomplicated, severe disease with central nervous system (CNS) involvement occurs in a small number of cases and is particularly linked to infection with EV-A71 or CVA6. Central nervous system (CNS) complications (most commonly meningoencephalitis or brainstem encephalitis) are seen in up to 30% of hospitalized cases of EV-A71 associated HFMD [4]. Severe cases can progress rapidly to cardiopulmonary failure which is the principal cause of death from HFMD [4].

There is a close temporal relationship between neurological signs and onset of tachycardia, hypertension and pulmonary oedema in those with severe disease. Magnetic resonance imaging (MRI) features, mainly studied in EV-A71 disease, show inflammation in the grey matter in the spinal cord, hypothalamus and medulla oblongata, including the dorsal nucleus of the vagus nerve [2, 5]. Consequently, it is hypothesized that cardiorespiratory complications of severe HFMD are linked to autonomic nervous system dysregulation resulting from CNS damage [2].

Clinical features suggestive of autonomic nervous system activation may be early indicators of those at risk of progression to severe diseases [6]. Nevertheless, these features lack specificity limiting their use, particularly in outbreak situations. Heart rate variability (HRV), is an alternative indicator of autonomic nervous system activity. HRV parameters are derived from statistical analysis of beat-to-beat variation in the heart rate. From these, inferences are drawn regarding the activity of the autonomic nervous system, normally responsible for control of heart rate (Supplementary Table 1) [7]. In a study of 46 Taiwanese children with clinical signs of HFMD and herpangina [8, 9], a reduction in HRV with increasing disease severity was observed. Early changes in HRV, preceding clinical signs, were observed in the 6 cases who progressed to severe disease.

Whilst promising as a tool for triage and detection of children at risk of severe disease, traditional HRV evaluation is conducted retrospectively from Holter monitor ECG recordings. Not only is this cumbersome in the small children in whom most HFMD occurs, but it is also unsuitable for real-time analysis needed in for clinical utility. The advent of light-weight wearable ECG monitors raises the possibility of using such devices to evaluate HRV changes in real-time [10, 11]. Such systems, in theory, are ideally suited for outbreak situations in low-resource settings as many are low-cost and potentially scalable, but the feasibility of this approach has yet to be established [12].

The aim of this study was to pilot the use of low-cost wearable devices to record ECG in children with HFMD, evaluating feasibility of use as well as describing HRV characteristics related to the underlying viral aetiology and clinical severity.

## Methods

The study was conducted at the Children’s Hospital 1, a tertiary care center located in Ho Chi Minh City which provides child’s health services for children up to 16 years old in Southern Vietnam. Hospitalized patients aged 16 years old or younger, with a clinical diagnosis of HFMD of Grades 2a to grade 4 (Table 1 [13]) during the first 7 days of illness were eligible for enrollment. Patients’ legal representatives gave written informed consent before patients were enrolled. As this was a pilot study, a pragmatic sample size of at least 120 patients was calculated in order to include at least 30 very severe patients (Grade 3 or 4) [14].

**Table 1:**
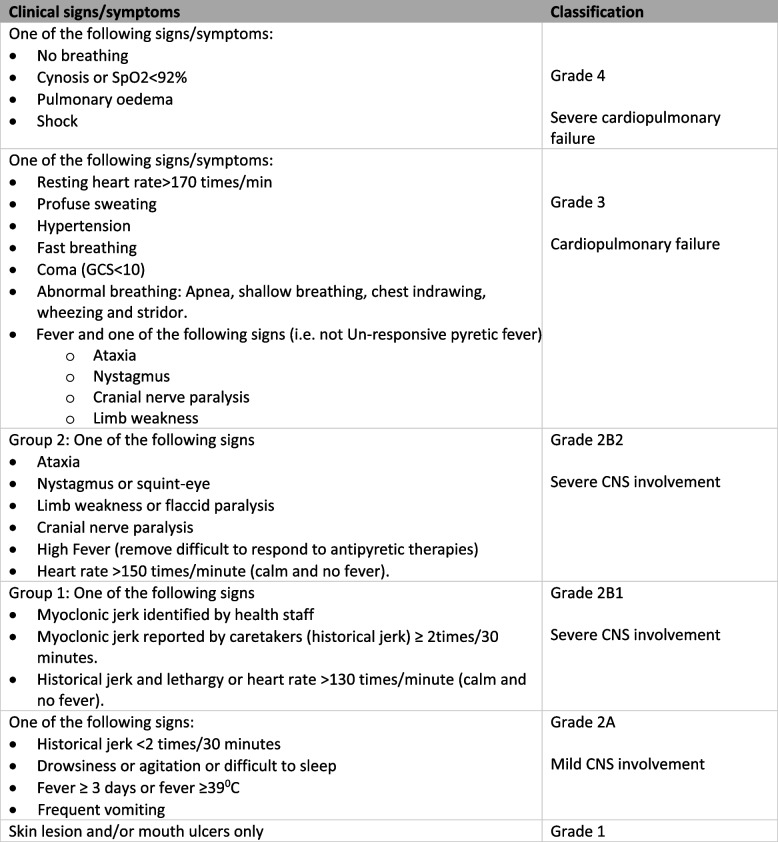
Viet Nam Ministry of Health Classification of hand foot and mouth disease [13]

ECG signal was collected for up to 24 h, using e-Patch (DELTA Danish Electronics) wearable ECG monitor (See Supplementary Fig. 1). Patient’s clinical and demographic data were collected at enrolment and patients followed until hospital discharge. Throat swabs were collected from all participants at enrolment in order to identify pathogens using PCR methods previously described [3]. Normal values for heart rate are given in Supplementary Box 1.

HRV analysis was performed after manually editing of ECG to exclude noise and artefacts. Recordings were divided into 5-min windows. All 5-min windows with artefacts or noise were excluded. The remaining recordings were analyzed using the sequential function of HRV analysis software version 1.1 (St. Etienne University, France [15]). For severe and very severe patients (Grades 2b, 3 and 4) who were admitted to the Emergence Room or Intensive Care Unit, the mean HRV indices derived from night time windows (between 10 pm to 4 am) were used for analysis as these were likely to be times of maximum quiet. For children with mild grades who were observed in other wards, recording durations were approximately six hours during day-time. For these patients, means of HRV indices from three consecutive five-minute HRV windows when patients were calm (based on regularity of heart rates) were used.

HRV indices were calculated with the reference being standard and a re-sampling rate of 2 Hz. HRV was analyzed using time domain, frequency domain and non-linear (Poincaré plot analysis) approaches [16]. The HRV indices selected are defined in Supplementary Table 1 and were selected as most commonly used and those recommended by consensus guidelines.

Median and inter quartile range (IQR) are given for skewed variables. Statistical differences in HRV indices among severities of disease and groups of detected pathogens were tested using the Mann–Whitney U- test. Corrections were not made for multiple testing.

## Results

From April 2017 to December 2018, 142 patients were enrolled in the study. Of these, the numbers of children with grade 2a, 2b1, 2b2, grade 3 and grade 4 were 40 (28.2%), 16 (11.3%), 32 (22.5%), 50 (35.2%) and 4 (2.8%) respectively. Demographic characteristics and clinical features at enrollment are described in Table 2. Almost all cases (97.2%) were younger than 60 months with a median age of 21.7 (IQR: 2.4–154.8) months. Of the patients enrolled, 34 (23.9%) were treated with phenobarbital, 2 (2.1%) with vasopressors/inotropes and 33 (23.2%) with milrinone as per Ministry of Health guidelines [13]. Two patients required mechanical ventilation and all patients survived to hospital discharge.

**Table 2:** Baseline characteristics of patients with HFMD on admission

Variables	Mild (Grade 2a) *n* = 40	Severe (Grades 2b1&2b2) *n* = 48	Very severe (Grades 3&4) *n* = 54	Total *n* = 142
*Demographics*				
**Male/female**	21/19	26/22	35/19	82/60
**Median age (months)** **(range)**	16 (2.4–43.2)	25.6 (6.8–154.8)	19 (4.1–118.2)	21.7 (2.4–154.8)
**HCM city origin**	18 (45)	19 (39.6)	23 (42.6)	60 (42.3)
**Other provinces**	22 (55)	29 (60.4)	31 (57.4)	82 (57.7)
**Length of stay (days) Median (range)**	3 (2–10)	4 (2–10)	7 (1–43)	5 (1–43)
*Clinical features (n, %)*				
**Skin lesion**	37 (92.5)	45 (93.8)	54 (100)	135 (95.1)
**Mouth ulcer**	38 (95)	45 (93.8)	46 (85.2)	129 (90.8)
**High fever**	25 (62.5)	36 (75)	50 (92.6)	111 (78.2)
**Cough**	4 (10)	2 (4.1)	6 (11.1)	12 (8.5)
**Runny nose**	13 (32.5)	1 (2.1)	3 (5.5)	17 (12)
**Vomiting**	11 (27.5)	11 (22.9)	8 (14.8)	30 (21.1)
**Diarrhea**	4 (10)	6 (12.5)	8 (14.8)	18)12.7)
**Lethargy**	9 (22.5)	34 (70.8)	41 (75.9)	84 (59.2)
**Irritable**	35 (87.5)	39 (81.3)	38 (70.4)	111 (78.2)
**Myoclonus (history)**	8 (20)	35 (72.9)	33 (61.1)	75 (52.8)
**Sweating**	2 (5)	1 (2.1)	5 (9.2)	8 (5.6)
**Drowsiness**	1 (2.5)	3 (6.3)	10 (18.5)	14 (9.9)
**Trembling**	0	18 (37.5)	16 (29.6)	34 (23.9)
**Mottled skin**	0	2 (4.1)	5 (9.2)	7 (4.9)
**Pulse (beats/minute)** **Median (range)**	140 (110–168)	140 (106–187)	148 (118–190)	143.5 (106–190)
**SBP (mmHg)** **Median (range)**	92.5 (80–110)	100 (90–160)	105 (85–160)	100 (80–160)
**DBP (mmHg)** **Median (range)**	60 (45–68)	60 (50–95)	60 (52–100)	60 (45–100)
**Respiratory rate (breaths/minute)** **Median( range)**	32 (22–60)	34 (22–46)	38 (22–70)	34 (22–70)
*Pathogens (n, %)*				
**EV-A71**	12 (30)	31 (64.6)	37 (68.5)	80 (56.3)
**CVA6**	2 (5)	2 (4.3)	1 (1.8)	5 (3.5)
**CVA10**	6 (15)	0	0	6 (4.2)
**CV-A16**	2 (5)	3 (6.4)	2 (3.6)	7 (4.9)
**Other enterovirus serotypes**^**a**^	8 (20)	4 (8.3)	8 (14.8)	20 (14.1)
**PCR negative**	10 (25)	7 (14.6)	7 (13)	24 (16.9)

^a^Consists of CV-A5 (1 case), CV-A8 (1 case) and unserotype enteroviruses (18 cases)

In all patients, ECG signal was obtained and no adverse events associated with the wearable occurred. A total of 5526 5-min ECG recordings were available for analysis. Of these, 120 segments were from patients with grade 2a disease, 863 from grade 2b1, 1783 from 2b2, 2627 from grade 3 and 133 from children with grade 4 disease.

Clinical features on admission are given in Table 2. As may be expected, clinical presentation, the proportion of high fever, lethargy, myoclonus, trembling and mottled skin in severe and very severe group were more prominent than in the mild group. The most common enterovirus serotype was EV-A71 80/142 (56.3% of cases). In the severe and very severe group, 68/102 (66.7%) children had EV-A71 detected. Conversely, CVA6, CVA10 and CVA16 were more common in those with mild disease (Table 2).

**Fig. 1:**
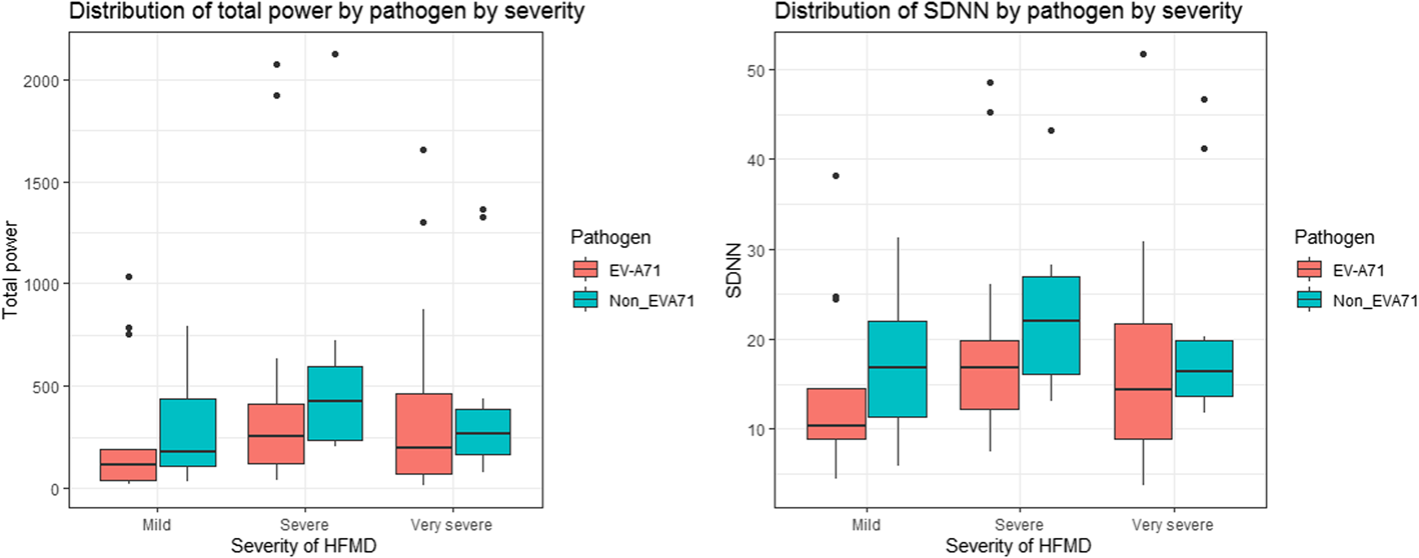
HRV indices according to both disease severity and pathogen. Figure shows HRV indices according to pathogen (EVA-71 red, other viruses green) and severity of disease. Mild (2a), Severe (2b1), very severe (2b2, 3 & 4). Panels: left Total power (ms.^2^); right Standard deviation of NN intervals -SDNN (ms)

**Fig. 2:**
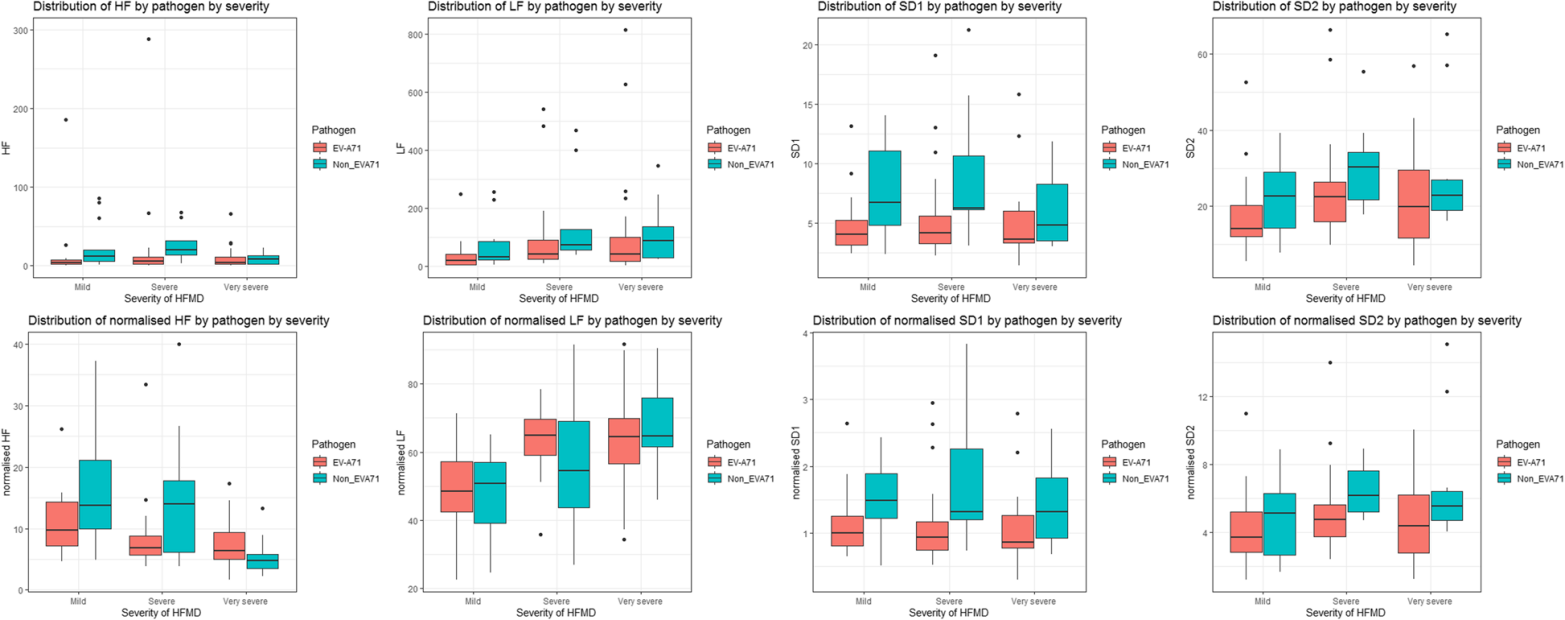
Distribution of HRV indices reflecting the general activity of ANS by pathogens in different severities. Figure shows HRV indices according to pathogen (EVA-71 red, other viruses green) and severity of disease. Mild (Grade 2a), Severe (Grades 2b1& 2b2) very severe (Grades 3 & 4). Panels above-below from left to right: High Frequency (HF) (ms^2^); High frequency normalized units (HFnu) (%); Low Frequency (LF) (ms.^2^); Low Frequency normalized units (LFnu) (%); SD1 (ms); SD1 normalizised units (%); SD2 (%); SD2 (ms); SD2 normalized units (%)

HRV data are presented in Figs. 1 and 2 and Supplementary Tables 2 and 3. When comparing HRV parameters according to pathogen, HRV parameters in those with EVA-71 associated disease were consistently lower across and severities (Figs. 1 and 2). Those parameters associated with overall ANSD activity shown in Fig. 1 show a trend of reduced measured variability in more mild disease (Supplementary Table 3). Specifically looking at variability parameters linked to ANSD activity and balance (Fig. 2), high frequency (HF) variability which is associated with vagal activity is lower with increasing disease severity, particularly in EVA-71 disease. The opposite pattern is seen with low frequency (LF) measurements, generally accepted to represent a combination of sympathetic and parasympathetic activity. SD1 and SD2 values (indicating parasympathetic and parasympathetic/sympathetic activity respectively) are lower in EVA-71 patients but trends related to disease severity are less clear.

## Discussion

We have demonstrated the feasibility of continuous ECG monitoring using wearable devices in young children with HFMD. Unlike traditional methods of wired monitoring or Holter ECG monitoring, wearable devices offer the ability to monitor patients of varying disease severity using a single device. The device we chose has the advantage of being a two-part system with a re-usable sensor connected to a disposable single-use electrode and is thus particularly suited for use in resource-constrained settings.

Interpreting HRV in detail in this study is complex due to the observational nature of our study and complex nature of cardiovascular control itself. Nevertheless, our findings regarding HRV values are consistent with other studies demonstrating lower values than healthy children and a relationship with disease severity [9, 17, 18]. We did not include patients with Grade 1 disease as these patients are not admitted to hospital and a future study may also include this group. Unlike previously reported HRV data for HFMD, we have been able to describe HRV according to specific pathogen and have demonstrated reduced HRV with EVA-71 associated disease, supporting clinical and imaging findings that EVA-71 is associated with most severe disease and CNS involvement [1].

The HRV metrics we present include a mixture of time, frequency and non-linear evaluations of beat-to-beat heart rate variation occurring in 5-min segments. Our data show reassuring internal consistency between time, frequency and non-linear measures. Clinical interpretation of individual indices, however, is complex. Clearest evidence supports interpretation of high-frequency HRV as representing vagal activity whereas other measures include more complex interactions between parasympathetic, sympathetic and vasomotor reflexes [8]. Nevertheless, a general interpretation of our data suggest a relative increase in sympathetic activity compared to parasympathetic and a reduction in parasympathetic activity in more severe disease or disease with EVA-71. A similar conclusion was made by authors of the study in Taiwanese children and is supported by MRI data of medulla oblongata involvement [2, 19].

Given the complex nature of HRV and the pragmatic nature of our pilot study, we caution over-interpretation of our data. HRV can be affected by eating, exercise, emotional state and drugs, all of which may be different between the groups in our study. Furthermore, more severe cases remained in hospital for longer periods giving rise to further possible bias in our results. By using only 5-min segments, we achieved a consistent length window for HRV analysis as window duration can significantly affect some indices. Data from a larger sample may allow better control for confounding factors and we believe that wearable devices may offer a suitable collection method for further data. With appropriate technology, data can be fed into near real-time decision support tools and assist in prognostic models for clinical care and triage. Currently there are still limited platforms and software available for such prognostication, especially in LMICs. In parallel with this project, we have created an open-source signal quality control tool and are working with a local partner to create a scalable real-time platform. However such initiatives are rare and without appropriate support from commercial partners or funders, innovative technologies are at risk of being developed only for high-income settings and problems [20–24].

## Conclusions

Our overall aim in this study was to pilot the use of wearables and gain some insight into pathogen differences in HRV. Our results indicate that wearable monitors are feasible in young children in a LMIC setting and that there are differences in HRV related to disease aetiology. These differences could potentially be incorporated into prognostic models to aid triage in outbreak situations.

### Supplementary Information

**Additional file 1: Supplementary Table 1.** Commonly used heart rate variability indices. **Supplementary Box 1.** Children’s Hospital 1 guidelines for normal heart rate in children within study age-groups. **Supplementary Table 2.** Heart rate variability indices in children with HFMD by pathogen. Definitions of HRV indices are given in Supplementary Table 1. **Supplementary Table 3.** HRV indices according to grade of disease. Definitions of HRV indices are given in Supplementary Table 1.

**Additional file 2.**

## Data Availability

The datasets used and/or analysed during the current study are available from the corresponding author on reasonable request.
