# Supplementary material for: Feasibility of wearable monitors to detect heart rate variability in children with hand, foot and mouth disease
Source: BMC Infect Dis. 2024 Feb 15;24:205. doi: 10.1186/s12879-024-08994-x (PMC10868055; doi:10.1186/s12879-024-08994-x)
Supplement: Supplementary file 1 — Additional file 1: Supplementary Table 1. Commonly used heart rate variability indices. Supplementary Box 1. Children’s Hospital 1 guidelines for normal heart rate in children within study age-groups. Supplementary Table 2. Heart rate variability indices in children with HFMD by pathogen. Definitions of HRV indices are given in Supplementary Table 1. Supplementary Table 3. HRV indices according to grade of disease. Definitions of HRV indices are given in Supplementary Table 1. [file 12879_2024_8994_MOESM1_ESM.docx]

**Supplementary Table 1 Commonly used heart rate variability indices (**Task Force of the European Society of Caridiology and the North American Society of Pacing Electrophysiology. Hear rate variability. Circulation. 1996;1043–65)

| HRV indices | Definition | Median (18)  (3^rd^-97^th^ percentile) |
| --- | --- | --- |
| *Time domain* | |  |
| HR (beats/minute) | Heart rate | 105.3 (88.5-130.6) |
| Standard deviation of NN intervals -SDNN (ms) | Standard deviation of all NN intervals, a global of RR intervals variability, reflects an estimate of short-term beat-to-beat variability | 73.4 (32.7-118.5) |
| Square root of  the mean squared differences of successive NN intervals - RMSSD (ms) | The square root of the mean of the sum of the squares of differences between adjacent NN intervals. This index measures short-term beat-to-beat variability and is associated with parasympathetic activity | 14.6 (41.4-127.3) |
| *Frequency domain* | |  |
| Total power – P_tot (ms^2^) | Five- minute total power is the variance of RR intervals over the temporal segment (<4Hz) | 4716 (1061-19692) |
| Very low frequency power - VLF (ms^2^) | Power in very low frequency range (≤0.04 Hz). Activity is abolished with atropine and therefore likely to represent parasympathetic activity | 938 (267-2517) |
| Low frequency power-LF (ms^2^) | Power in low frequency range (0.04 Hz-0.15 Hz) Modulated by the baroreflex and representing sympathetic and parasympathetic activity | 648 (122-2723) |
| LF nu (%) | LF power in normalized units; LF/(Total power –VLF)*100 |  |
| High frequency power-HF (ms^2^) | Power in high frequency range (0.15-0.4 Hz) representing parasympathetic (vagal activity), although can be affected by very high or low respiratory rates | 700.1 (73-8508.2) |
| HF nu (%) | HF power in normalized units; HF/(Total power –VLF)*100 |  |
| LF/HF | Ratio LF/HF | 1.30 (0.50-3.33) |
| *Poincaré plot* | |  |
| Standard deviation 1 (Width of ellipse) - SD1 (ms) | Standard deviation of the distance of each point from the y = x axis. This is an index from Poincaré plot that represents short-term RR intervals variability. It is mediated by the parasympathetic nervous system via controlling the sinus node | 26.51 (8.98-77.60) |
| Standard deviation 2 (Length of ellipse) - SD2 (ms) | Standard deviation of the distance of each point from the *y* = *x* + average R–R interval. This is an index from Poincaré plot that represents long-term RR intervals variability. It reflects both parasympathetic and sympathetic activity | 123.84 (61.92-198.6) |

**Supplementary Box 1:** Children’s Hospital 1 guidelines for normal heart rate in children within study age-groups

| 1. Newborn (up to one month): 70-180 beats per minute. 2. one month to 11 months: 80-160 beats per minute. 3. one to two years old: 80-140 beats per minute. 4. three to 4 years old: 80-120 beats per minute |
| --- |

**Supplementary Table 2** Heart rate variability indices in children with HFMD by pathogen. Definitions of HRV indices are given in Supplementary Table 1

| HRV indices | EV-A71 (n=80)  Median (IQR) | Non-EV-A71 (n=38)  Median (IQR) | P-value |
| --- | --- | --- | --- |
| Heart rate (times/minute) | 137.88 (128.01 - 144.59) | 130.08 (118.91 - 145.99) | 0.188 |
| SDNN (ms) | 14.38 (9.24 - 21.29) | 17.38 (12.64 - 23.51) | 0.070 |
| RMSSD (ms) | 5.72 (4.67 - 8.50) | 8.77 (5.26 - 13.76) | 0.001 |
| Ptot (ms^2^) | 194.57 (85.99 - 476.32) | 286.15 (122.62 - 544.19) | 0.186 |
| VLF (ms^2^) | 116.27 (44.11 - 313.64) | 147.89 (52.52 - 260.00) | 0.609 |
| LF (ms^2^) | 40.71 (19.29 - 86.86) | 59.11 (28.87 - 115.62) | 0.103 |
| HF (ms^2^) | 4.31 (1.74 - 10.89) | 12.31 (2.75 - 21.49) | 0.004 |
| LFnu (%) | 64.32 (54.10 - 69.36) | 55.03 (42.79 - 64.86) | 0.008 |
| HFnu (%) | 7.17 (5.54 - 9.71) | 11.62 (5.60 - 17.65) | 0.012 |
| LF/HF | 10.67 (7.57 - 15.08) | 5.00 (2.79 - 13.89) | 0.001 |
| SD1 (ms) | 4.02 (3.22 - 5.98) | 6.20 (3.71 - 9.74) | 0.001 |
| SD2 (ms) | 19.95 (12.59 - 27.82) | 23.32 (16.22 - 31.90) | 0.090 |
| SD1/SD2 | 0.25 (0.20 - 0.32) | 0.31 (0.21 - 0.44) | 0.130 |
| SD1nu (%) | 0.93 (0.76 - 1.26) | 1.32 (0.98 - 1.90) | 0.001 |
| SD2nu (%) | 4.55 (2.98 - 5.81) | 5.23 (3.85 - 6.46) | 0.122 |

**Supplementary Table 3**: HRV indices according to grade of disease

Definitions of HRV indices are given in Supplementary Table 1

| HRV indices  Median (IQR) | Mild  Grade 2a  (n=40) | Severe  Grade 2b1/2b2  (n=48) | Very severe  Grade 3/4  (n=53) |
| --- | --- | --- | --- |
| Heart rate (beats/minute) | 125.53 (117.76-143.42) | 137.23 (124.25-142.99) | 137.88 (127.61-147.56) |
| SDNN (ms) | 12.37 (9.50-20.97) | 16.82 (12.78-21.84) | 14.38 (9.31-21.77) |
| RMSSD (ms) | 8.46 (4.93-12.88) | 6.45 (4.69-9.25) | 5.43 (4.72-8.61) |
| Ptot (ms^2^) | 128.00 (72.20-351.28) | 256.38 (142.97-486.49) | 189.13 (76.34-445.02) |
| VLF (ms^2^) | 71.78 (30.44-188.96) | 158.97 (99.91-306.12) | 116.27 (41.11-266.51) |
| LF (ms^2^) | 29.76 (15.17-63.80) | 48.41 (25.59-104.53) | 42.53 (18.37-113.15) |
| HF (ms^2^) | 6.38 (2.96-24.60) | 6.35 (2.60-18.01) | 3.59 (1.78-11.89) |
| LFnu (%) | 48.28 (37.70-60.27) | 63.76 (53.11-69.30) | 64.38 (54.72-78.65) |
| HFnu (%) | 13.30 (9.09-19.79) | 7.35 (5.73-11.82) | 6.02 (4.30-8.71) |
| LF/HF | 3.85 (2.56-6.15) | 11.31 (5.86-15.66) | 12.56 (9.09-18.58) |
| SD1 (ms) | 5.99 (3.49-9.11) | 4.51 (3.31-6.54) | 3.73 (3.33-6.09) |
| SD2 (ms) | 15.75 (12.33-27.51) | 22.53 (16.90-29.95) | 19.95 (12.74-29.52) |
| SD1/SD2 | 0.34 (0.27-0.49) | 0.25 (0.20-0.32) | 0.24 (0.18-0.31) |
| SD1nu (%) | 1.24 (0.89-1.83) | 1.04 (0.76-1.37) | 0.94 (0.79-1.33) |
| SD2nu (%) | 3.71 (2.56-5.57) | 4.97 (3.90-6.06) | 5.04 (3.06-6.46) |
