# Supplementary figures and images for: Feasibility of wearable monitors to detect heart rate variability in children with hand, foot and mouth disease
Source: BMC Infect Dis. 2024 Feb 15;24:205. doi: 10.1186/s12879-024-08994-x (PMC10868055; doi:10.1186/s12879-024-08994-x)

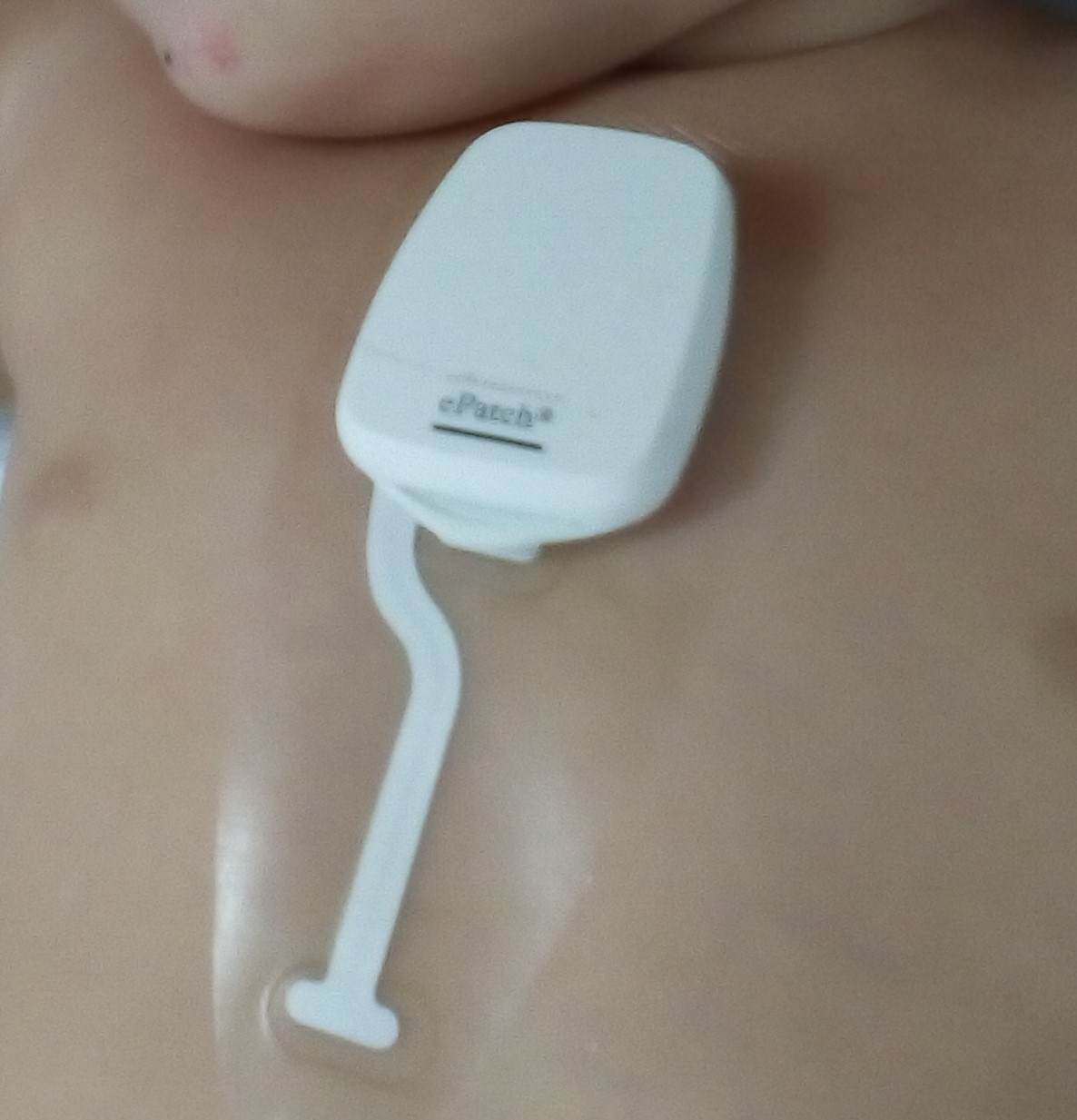

Supplement: Supplementary file 2 — Additional file 2. [file 12879_2024_8994_MOESM2_ESM.jpg]
